# Antagonistic effects of MYC and hypoxia in channeling glucose and glutamine into *de novo* nucleotide biosynthesis

**DOI:** 10.1186/2049-3002-2-S1-O10

**Published:** 2014-05-28

**Authors:** Teresa Fan, Anne Le, Zachary Stine, Ye Yang, Karen Zeller, Weiqiang Zhou, Hongkai Ji, Richard Higashi, Chi Dang, Andrew Lane

**Affiliations:** 1 Graduate Center for Toxicology and Markey Cancer Center, University of Kentucky, Lexington, KY, USA; 2 Department of Pathology and Oncology, School of Medicine, Johns Hopkins University, Baltimore, MD 21231, USA; 3 Abramson Cancer Center, University of Pennsylvania, Philadelphia, PA 19104, USA; 4 Department of Medicine, School of Medicine, Johns Hopkins University, Baltimore, MD 21231, USA; 5 Department of Biostatistics, Bloomberg School of Public Health, Johns Hopkins University, Baltimore, MD 21231, USA

## Background

Cell proliferation requires up regulation of nucleotide biosynthesis for making new DNA and RNA to support protein bio-synthesis. MYC is a major transcription factor that regulates metabolic processes essential for cell division, and is overexpressed in many cancers. The nutrient sources and integration of the metabolic pathways for nucleotide biosynthesis that enable MYC-dependent cell division are poorly defined. Using Stable Isotope Resolved Metabolomics (SIRM) we have determined the fate of atoms from ^13^C_6_-glucose, ^13^C_5_,^15^N_2_-glutamine, or ^2^H-glycine into nucleotides under varied conditions of MYC expression in the *MYC*-inducible P493-6 B-lymphocyte [2] and several lung cell lines.

## Materials and methods

P493-6 cells, A549, PC9 and HPLD1 cells were grown in the presence of [U-^13^C]-glucose, [U-^13^C,^15^N]-Gln or [^2^H_2_]-Gly for one cell division. P493-6 cells, which have an inducible MYC gene were grown under four sets of conditions, namely MYC On/Off under 21% or 1% oxygen. Media samples were taken at timed intervals, and the cells were harvested, and extracted. Isotopomer and isotopologue distributions were measured in the free nucleotides and in metabolites that characterize the related pathways of glycolysis, PPP, Krebs cycle, serine, glutathione and nucleobase biosynthetic pathways. In parallel, gene expression data and MYC promoter occupancies were interrogated.

## Results

MYC increased incorporation of ^13^C from glucose and glutamine into newly synthesized glycine and aspartate, which are channeled into nucleotides. MYC suppression in lung adenocarcinoma PC9 and A549 cells induced opposite effects on carbon flow into nucleotides. Exogenous ^2^H-Glycine was preferentially incorporated into glutathione both in transformed cells and primary lung HPLD1 cells, whereas glucose-derived ^13^C Gly was incorporated into purines. Although hypoxia enhances glycolysis and maintains ribose synthesis in P493 cells, it antagonizes channeling toward nucleobase synthesis and reduces MYC-induced proliferation. MYC increased coordinately expression of the relevant metabolic genes associated directly and indirectly with nucleotide biosynthesis, which correlates with cell proliferation and cell cycle distribution data.

## Conclusions

These results reveal the coupling of bioenergetics and nutrient availability to cell proliferation through regulation of metabolic channeling in de novo nucleotide biosynthesis. MYC coordinately regulates genes for nucleotide biosynthesis as well as the associated pathways for serine and glycine synthesis, which feed into the purine pathway. MYC enhances channeling of glucose-derived Gly into purines and Gln-derived Asp into pyrimidines.

